# Systematic analysis of long non-coding RNA and mRNA expression changes in ApoE-deficient mice during atherosclerosis

**DOI:** 10.1007/s11010-019-03610-y

**Published:** 2019-08-24

**Authors:** Xiaoqian Lou, Xiaoyan Ma, Dawei Wang, Xiangjun Li, Bo Sun, Tong Zhang, Meng Qin, Liqun Ren

**Affiliations:** 1 Department of Experimental Pharmacology and Toxicology, School of Pharmacy, Jilin University, Changchun, 130021 Jilin People’s Republic of China; 2 Department of Endocrinology, The First Hospital of Jilin University, Changchun, 130021 Jilin People’s Republic of China; 3 Department of Cardiology, The Affiliated Hospital of Changchun University of Chinese Medicine, Changchun, 130021 Jilin People’s Republic of China; 4 Department of Emergency, The First Hospital of Jilin University, Changchun, 130021 Jilin People’s Republic of China

**Keywords:** LncRNA, mRNA, Atherosclerosis, ApoE-deficient mouse

## Abstract

Atherosclerosis plays an important role in the pathology of coronary heart disease, cerebrovascular disease, and systemic vascular disease. Long non-coding RNAs (lncRNAs) are involved in most biological processes and are deregulated in many human diseases. However, the expression alteration and precise role of lncRNAs during atherosclerosis are unknown. We report here the systematic profiling of lncRNAs and mRNAs in an ApoE-deficient (ApoE^−^/^−^) mouse model of atherosclerosis. Clariom D solutions for the mouse Affymetrix Gene Chip were employed to analyze the RNAs from control and ApoE^−^/^−^ mice. The functions of the differentially expressed mRNAs and lncRNAs and the relationships of their expression with atherosclerosis were analyzed by gene ontology, co-expression network, pathway enrichment, and lncRNA target pathway network analyses. Quantitative real-time PCR (QRT-PCR) was used to determine the expression of mRNAs and lncRNAs. A total of 2212 differentially expressed lncRNAs were identified in ApoE^−^/^−^ mice, including 1186 up-regulated and 1026 down-regulated lncRNAs (|FC| ≥ 1.1, *p* < 0.05). A total of 1190 differentially expressed mRNAs were found in the ApoE^−^/^−^ mice with 384 up-regulated and 806 down-regulated (|FC| ≥ 1.1, *p* < 0.05). Bioinformatics analyses demonstrated extensive co-expression of lncRNAs and mRNAs and concomitant deregulation of multiple signaling pathways associated with the initiation and progression of atherosclerosis. The identified differentially expressed mRNAs and lncRNAs as well as the related signaling pathways may provide systematic information for understanding the pathogenesis and identifying biomarkers for the diagnosis, treatment, and prognosis of atherosclerosis.

**Electronic supplementary material:**

The online version of this article (10.1007/s11010-019-03610-y) contains supplementary material, which is available to authorized users.

## Introduction

Atherosclerosis is one of the major diseases endangering human health worldwide and contributes to many human diseases with high mortality and disability rates, including coronary heart disease, stroke, and atherosclerosis of lower extremities [1–3]. Atherosclerosis is a natural process, but it may be accelerated in individuals with diabetes, hypertension, or hyperlipidemia. In the past few decades, great advances have been made in the diagnosis, prevention, and treatment of atherosclerosis; however, the precise molecular mechanisms underlying atherosclerosis remain largely unknown [4–9]. With the advent of next-generation sequencing, there is an urgent need to elucidate the genetic as well as epigenetic alterations that occur during the process of atherosclerosis.

Long non-coding RNAs (lncRNAs) are RNA transcripts longer than 200 nucleotides (nt) that lack open reading frames and protein-coding capabilities. lncRNAs affect numerous cellular processes by interacting with DNA, RNA, and proteins to regulate gene expression and chromatin dynamics [10–12]. lncRNAs play important roles in the initiation and progression of atherosclerosis and may serve as potential biomarkers for early diagnosis, potential therapeutic targets, and prognosis [13–16]. For example, lncRNA ANRIL was found to be associated with pathogenic changes in atherosclerotic plaques via the control of ribosomal RNA maturation and modulation of pathways regulating atherogenesis [17]. lncRNA MIAT was identified as a biomarker for myocardial infarction, which indicates its contribution to the pathogenesis of myocardial infarction [18]. However, the precise roles of lncRNAs in the pathogenesis of atherosclerosis are not yet fully understood. Therefore, there is an urgent need to further study the regulatory mechanisms of lncRNAs in atherosclerosis.

Although lncRNAs have been extensively studied in cancer as well as in vascular diseases, there is a lack of atherosclerosis animal models to dissect the differential expression of lncRNAs and their effects on the expression of protein-coding mRNAs. C57BL/6 mice are extensively used in the study of hyperlipidemia and atherosclerosis. Particularly, apolipoprotein E-deficient (ApoE^−/−^) mice have been widely used as animal models in the study of hyperlipidemia, atherosclerosis, non-alcoholic fatty liver, and its complications, as well as the physiological function of ApoE [19]. ApoE^−^/^−^ mice show abnormal hyperlipidemia and exhibit systemic metabolic and functional abnormalities including arterial fat accumulation after 12 weeks of high-fat diet feeding. In the present study, to investigate the differentially expressed lncRNAs in aortic tissue, we profiled the lncRNA and mRNA expression in three aortic tissue samples from ApoE^−^/^−^ mice and three aortic tissue samples from normal C57BL/6 mice using mRNA and lncRNA microarrays. Our study may provide fundamental resources to reveal the functions of lncRNAs during the progression of atherosclerosis.

## Materials and methods

### Animals

Three wild-type (male; age, 8 weeks old; weight, 26.63 ± 1.01 g) and three ApoE homozygous knockout (ApoE^−/−^) C57BL/6 J mice (male; age, 8 weeks old; weight, 27.24 ± 1.75 g) were purchased from Beijing HuaFuKang Bioscience Company. The mice were maintained in an animal facility at 22 ± 2 °C with a relative humidity of 60 ± 5%, under a 12-h light/dark cycle with free access to water at the Laboratory Animal Center. Wild-type C57BL/6 J mice (the control group) were fed with normal diet, while ApoE^−^/^−^ mice (the ApoE^−^/^−^ group) were fed with the Clinton high-fat diet with regular casein and 1.25% cholesterol (HFD, D12108C, Research Diets Inc). All procedures related to animals were performed according to the Chinese legislation on the use and care of laboratory animals and were approved by the ethics committee for laboratory animals of Jilin University (Changchun, China).

### Observation of atherosclerotic plaque

Mice were euthanized via breathing diethyl ether after the ApoE^−^/^−^ group was fed with HFD for 12 weeks, followed by perfusion with phosphate-buffered saline (PBS) through the left ventricle and fixation with 4% paraformaldehyde. The heart and aortic tissues were separated from the aortic root to the bifurcation of the iliac artery and fixed in 4% paraformaldehyde for 24 h. Oil red O staining was used to assess atherosclerotic plaque area and lipid accumulation in the aorta following the manufacturer’s protocol [20]. To assess the area of atherosclerotic lesions in the aortic region, the aorta was longitudinally dissected to expose the intimal surface, and the ratio of intimal atherosclerotic plaque area from the aortic root to iliac bifurcation was analyzed. Images were captured by a Canon EOS80D digital camera (Tokyo, Japan). Image-Pro Plus 6.0 software (NIH Image, USA) was applied to analyze the positive staining within the aortas. The red or orange areas were considered as positive regions. The area of an aortic atherosclerosis lesion was represented by the percentage of the oil red O positively stained area relative to the whole aortic intima area. None of the researchers who conducted the specimen collection and data collection and analysis were aware of the specific groups.

### RNA extraction and microarray analysis

After anesthesia via breathing diethyl ether, the animals were dissected, and the perfusion device was fixed by left atrial puncture. A solution of 0.9% saline was perfused until the residual blood in the cardiac cavity was washed out and then fixed by perfusion with polyformaldehyde. After multi-organ fixation, the heart and aorta were removed and separated along the descending aorta to the bifurcation of iliac artery. After washing with RNAse-free water, all tissue samples were transferred to a − 80 °C freezer within 10 min after resection for storage. Total RNA was extracted from the aortic tissue using TRIzol reagent (Life Technologies, Carlsbad, CA, USA) and purified with the RNeasy mini kit (Qiagen, Valencia, CA, USA) according to the manufacturers’ instructions. The quality and quantity of RNA were determined with a UV–Vis spectrophotometer (Thermo, NanoDrop 2000, USA) at 260 nm/280 nm absorbance. The lncRNA and mRNA transcription profiles of the samples were determined by Clariom D solutions for the mouse Affymetrix Gene Chip (Santa Clara, CA, USA). Microarray analysis was carried out by Cnkingbio (Beijing, China) and Certified by Affymetrix. The microarray analysis was presented using Affymetrix Expression Console Software (version 1.2.1). Primary data (CEL files) were normalized at the transcript level using the robust multi-array average method. The median summarization of transcript expressions was calculated according to the instructions. Signal scanning and analysis were performed using Affymetrix equipment (Affymetrix Gene Chip Operating Software).

### Bioinformatics analysis

#### Differential expression of mRNAs and lncRNAs

The random variance model (RVM) *t* test was applied to filter the differentially expressed genes between atherosclerotic lesion and normal aortic tissue according to a *p* value threshold. A *p* value < 0.05 was set as indicative of a significant difference. Then we conducted analysis of hierarchical clustering of lncRNAs and mRNAs with Cluster 3.0 software and TreeView v1.60. The data were normalized using the median summarization method (Cnkingbio, Beijing, China).

#### Gene ontology enrichment analysis

Gene Ontology (GO: http://www.geneontology.org) annotations were applied to figure out the functions of the differentially expressed mRNAs in aortic tissues of ApoE^−^/^−^ mice compared to normal aortic tissues. The results of GO analysis show the gene regulatory network of the differentially expressed genes in terms of molecular functions, biological processes, and cellular components.

#### Construction of co-expression network

A gene co-expression network was constructed to identify interactions among the differentially expressed lncRNAs and mRNAs based on a correlation analysis. The network was constructed according to the normalized signal intensities of specific mRNA and lncRNA expression levels where each node corresponds to a gene and a pair of nodes is connected with an edge if there is a significant co-expression relationship between them [21]. In principle, we followed a two-step approach: calculating a co-expression measure and selecting a significance threshold. Pearson’s correlation coefficients were used to identify lncRNAs and their coding genes [22]. The lncRNA–mRNA co-expression networks were constructed using Cytoscape software (The Cytoscape Consortium, San Diego, CA, USA).

#### Pathway network and lncRNA target pathway network

Pathway enrichment network analysis was performed to explore the significant pathways of the differentially expressed genes by using the Kyoto Encyclopedia of Genes and Genomes (KEGG; http://www.genome.jp/kegg/). Two-sided Fisher’s exact test and *χ*^2^ test were used to select significant pathways, and the threshold of significance was defined by a *p* value and false discovery rate (FDR). According to the interaction between pathways in the KEGG database, the interaction network between significant pathways with different genes was constructed, and the main distributions of significant pathways were determined. The core pathway group and its target genes in the network were analyzed and determined by graph theory. According to the differentially expressed lncRNAs and the significant pathways involved based on related target genes, the lncRNA target pathway network was constructed using the attributes of lncRNA and target gene pathways. The network values were calculated according to the location functions of lncRNAs in the network. lncRNAs with the highest Eigenvalue in the hub of the network had important regulatory value for multiple pathways. At the same time, by evaluating the Eigenvalues of pathways in the network, we could find the core pathways regulated by several different lncRNAs, thereby revealing important roles for the lncRNAs in the onset and progression of atherosclerosis.

#### Quantitative real-time PCR (QRT-PCR)

QRT-PCR was performed to verify the differential expression of lncRNAs and mRNAs between atherosclerotic plaque tissues of ApoE^−^/^−^ mice fed with a HFD for 12 weeks and normal aortic tissues. Total RNA extracted for microarray analysis was reverse-transcribed to cDNA using M-MLV reverse transcription (Promega, Madison, WI, USA) according to manufacturer’s instructions. QRT-PCR was performed using an ABI 7500 real-time PCR system (Applied Biosystems, Foster City, CA, USA) and a SYBR Green kit (Takara, Dalian, China) using the primer pairs listed in Table 1. We used 18S RNA as an endogenous control for normalization. For relative quantification, 2^−ΔΔCt^ was calculated and used as an indication of gene relative expression.

**Table 1:** Primer sequences used for qRT-PCR

Gene symbol	Forward	Reverse
Cldn1	ATCCACAGTCCCTCGTAG	GGTTTCATCCTGGCTTCT
Ccl8	ATACCCTGCTTGGTCTGG	GCTCATAGCTGTCCCTGTC
Spp1	ACCATGCAGAGAGCGAGGATT	GGGACATCGACTGTAGGGACG
Traf3	TCCGAGGTATCCACTATGA	TGTCGCCCAAACTGTTCT
Fas	TTCGTGAAACTGATAAAAACTGC	TCTGATGGTCTCCAAAATGCT
mapk3	GGCTTTCTGACGGAGTATGTGGC	TGGTCCAGGTAGTGCTTGCCG
NONMMUT000151	GCATCAATAGTCCCAGTAA	GATGGAGGTCTGGTTTGT
NONMMUT042398	GATCACTACAGCCTCCTC	CTGGTAAATCAGGCAACT
Gm13241	TTTGAAACCTCCTCCCTA	ATGCCCAGTGGTGTATTT
NONMMUT019491	ACCCTGCTTCTATTCCAC	GTAGGCAAACACCCAGAC
NONMMUT001754	CGAAATGAAGCGTAGTGT	TTGACAACCAGGAATAGC
NONMMUT051707	AACTAGGCTCACTGAATAAA	TGTAACTGGGAAGGAAGA

### Statistical analysis

All data were analyzed using SPSS 22.0 software (SPSS Inc., Chicago, IL, USA) and are shown as mean ± standard deviation. Student’s *t* test was used to compare lncRNAs and mRNAs between atherosclerotic plaque tissues and normal aortic tissues. The FDR rate was determined to correct the *p* value. Fold change (FC) was adopted to analyze the statistical significance of the microarray results. |FC| ≥ 1.1 and *p* < 0.05 were considered the critical values for designating differentially expressed lncRNAs and mRNAs.

## Results

### Atherosclerotic lesions are increased in the aorta of ApoE^−^/^−^ mice

**Fig. 1:**
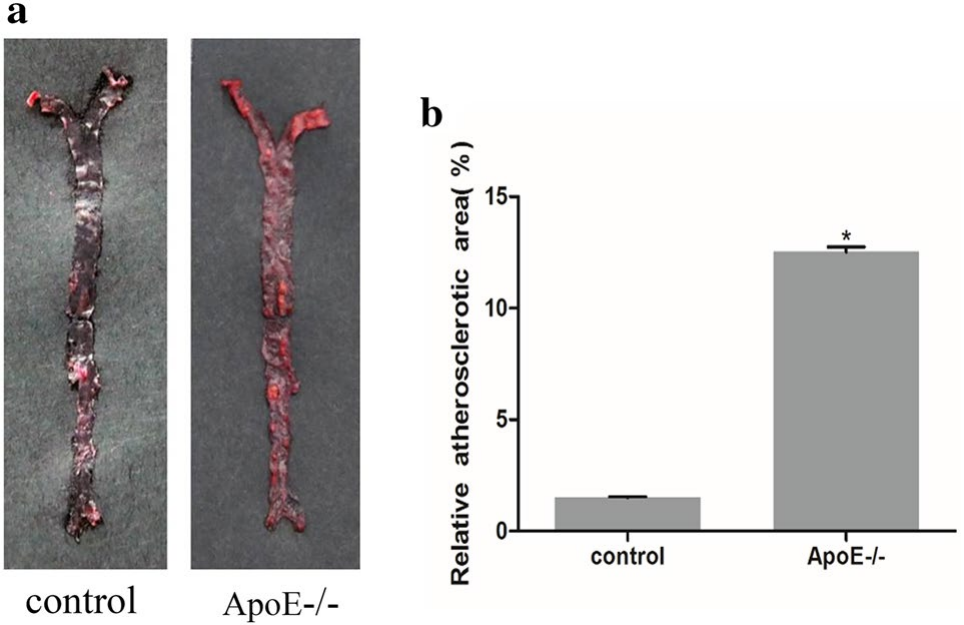
Atherosclerotic plaques were increased in the aortas of ApoE^−^/^−^ mice. **a** Oil red O staining of the aorta in wild-type control group and ApoE^−^/^−^ mice fed with HFD for 12 weeks. **b** The plaque area was quantified as a percentage of the oil red O stained area to the total aortic surface area. Numerical data are expressed as mean ± standard deviation of the mean (%). *p < 0.05

Oil red O staining showed significant accumulation of atherosclerotic plaques in the aortas of ApoE^−^/^−^ mice fed with a HFD for 12 weeks compared to that in normal control mice. Positive red stained areas, indicative of atherosclerotic plaques, were found throughout the aorta in ApoE^−/−^ mice, especially in the root of aorta, whereas there was much less positively stained area in the control group (Fig. 1a). Quantitative analysis demonstrated that the aortic atherosclerotic plaque area in ApoE^−^/^−^ mice was significantly higher than that in control mice (12% vs. 1%, *p* < 0.05).

### lncRNA and mRNA expression profiles in atherosclerotic plaque of ApoE^−^/^−^ mice

**Fig. 2:**
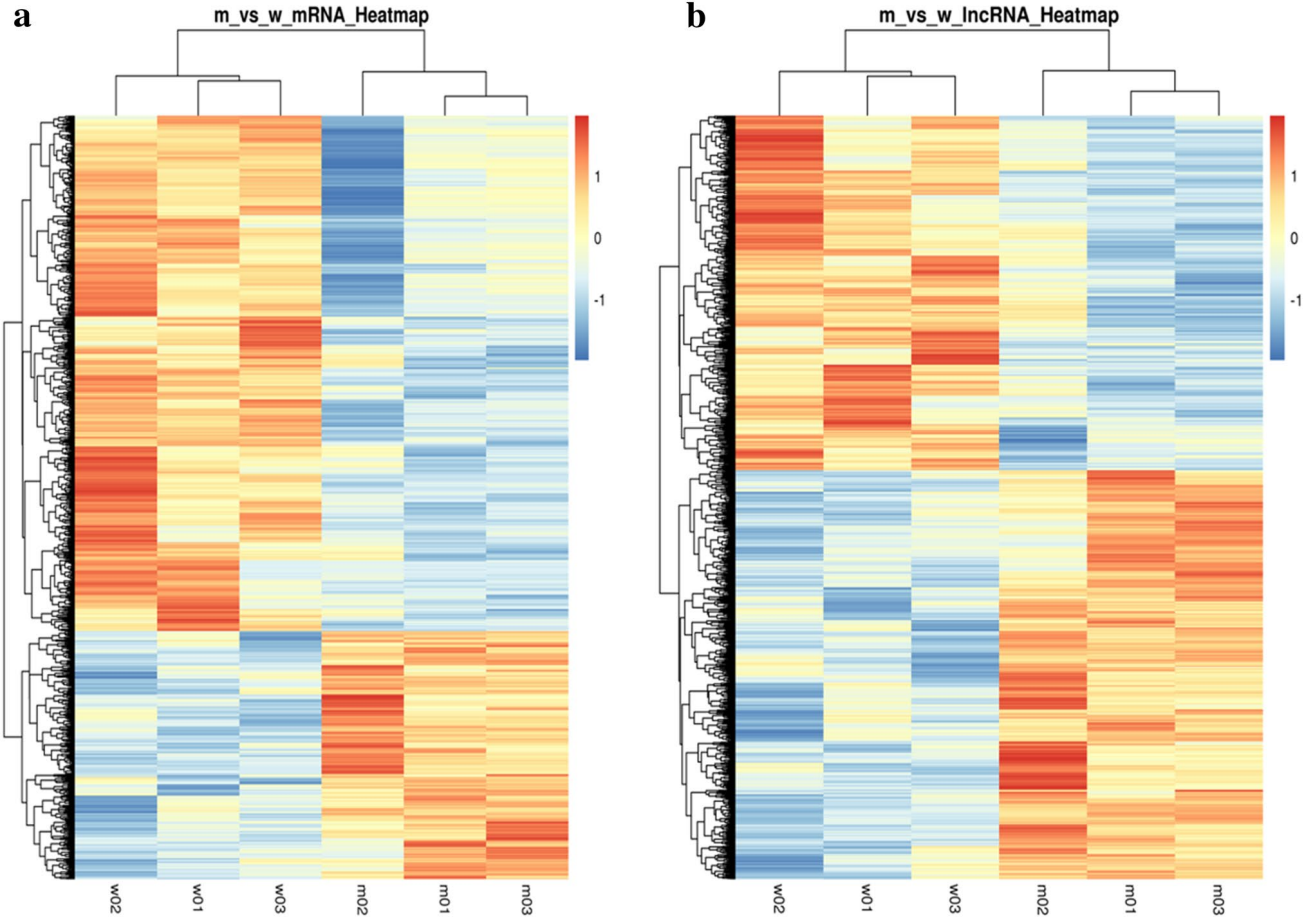
Hierarchical cluster analysis of mRNAs and lncRNAs in ApoE^−^/^−^ mice fed with HFD compared to the control group. **a** Differential expression of mRNAs (*p* < 0.05). **b** Differential expression of lncRNAs (*p* < 0.05). The relative gene log2 expression is expressed by a color gradient intensity scale. Blue color indicates down-regulation, and red color indicates up-regulation. Each row represents a single mRNA or lncRNA, and each column represents a separate sample. (Color figure online)

We performed microarray analysis to identify differentially expressed lncRNAs and mRNAs in ApoE^−^/^−^ mice relative to those in the control group. We identified 384 mRNAs that were up-regulated and 806 that were down-regulated in ApoE^−^/^−^ mice (|FC| ≥ 1.1, *p* < 0.05). The top five up-regulated and top five down-regulated genes are listed in Table 2. The most up-regulated gene was *SPP1* with a FC value of 12.22, while the most down-regulated gene was *CYP11B1* with a FC value of 22.84. Hierarchical clustering analysis of the variation of mRNA expression levels between ApoE^−^/^−^ mice and control group revealed distinct expression patterns in the two groups (Fig. 2a). A total of 2212 lncRNAs were identified as differentially expressed between ApoE^−^/^−^ and control mice including 1186 up-regulated and 1026 down-regulated lncRNAs (|FC| ≥ 1.1, *p* < 0.05). The top five up-regulated and top five down-regulated lncRNAs are listed in Table 3. The results of hierarchical clustering analysis of lncRNA expression patterns between ApoE^−^/^−^ mice and control group are shown in Fig. 2b.

**Table 2:** Top 10 differentially expressed mRNAs in the aorta between ApoE^−^/^−^ and control mice

Probe set	Gene symbol	FC	*p*	Alteration
TC1500001723.mm.1	CYP11B1	− 22.84726058	0.02508458	Down
TC1700002527.mm.1	SRD5A2	− 18.09243571	0.023230235	Down
TC0300002565.mm.1	HSD3B1	− 16.30418481	0.034709542	Down
TC0800000331.mm.1	STAR	− 15.41645187	0.022470665	Down
TC0900000722.mm.1	CYP11A1	− 14.54964851	0.028202292	Down
TC1000003214.mm.1	LILRB4	2.483170068	0.00810609	Down
TC1100001223.mm.1	CCL8	2.497324589	0.002006455	Up
TC0600003548.mm.1	IGKV4-90	2.708439648	0.029939868	Up
TC1400000317.mm.1	ITIH4	4.852895476	0.03408526	Up
TC0500001025.mm.1	SPP1	13.2216744	0.015462668	Up

**Table 3:** Top 10 differentially expressed lncRNAs in the aorta between ApoE^−^/^−^ and control mice

Probe set	Gene symbol	FC	*p*	Alteration
TC0300002563.mm.1	GM12431	− 15.58238	0.040804005	Down
TC1500001722.mm.1	NONMMUT023944	− 15.52936	0.034657601	Down
TC1700001922.mm.1	CYP21A2-PS	− 8.738724	0.03304234	Down
TC0600002382.mm.1	NONMMUT057179	− 5.930201	0.001186209	Down
TC1500001682.mm.1	KNOWTID_00002976	− 5.636188	0.025034281	Down
TC1700000139.mm.1	AB344273	2.3764656	0.003912766	Up
TC0500000822.mm.1	NONMMUT053025	2.4703776	0.021864737	Up
TC1400000740.mm.1	TRAJ1	2.698691	5.14976E−05	Up
TC0500000412.mm.1	NONMMUT051994	3.1559759	7.28004E−05	Up
TC0X00002558.mm.1	NONMMUT073071	4.1698495	0.023279728	Up

### GO enrichment

**Fig. 3:**
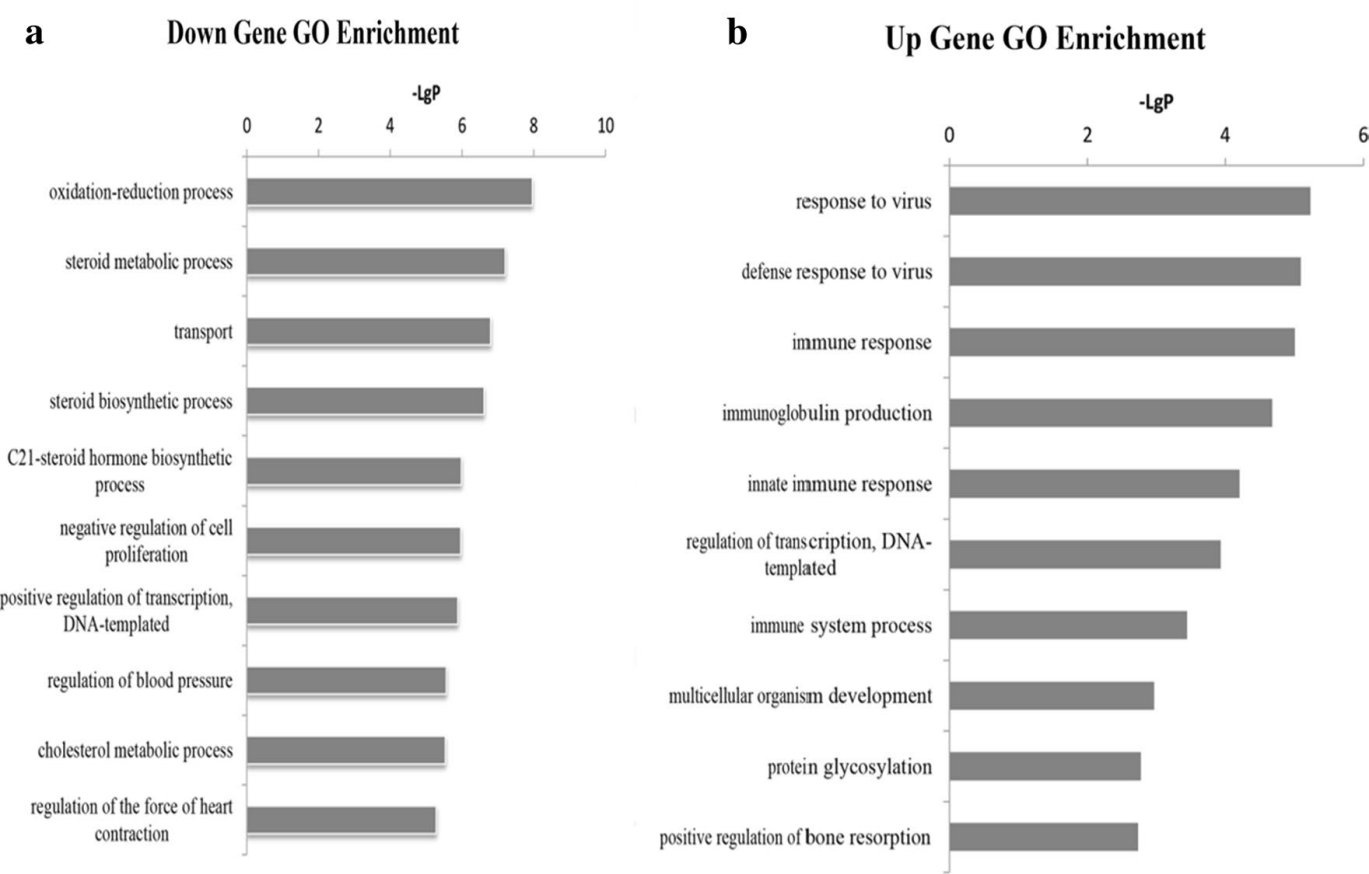
GO enrichment analysis of the altered mRNAs in ApoE^−^/^−^ mice. The vertical axis is the GO category, and the horizontal axis is the enrichment of GO that shows a significant difference in gene function. **a** GO enrichment analysis of down-regulated mRNAs. **b** GO enrichment analysis of up-regulated mRNAs

GO analysis was explored to evaluate the differentially expressed mRNAs in biological processes, molecular functions, and cellular components. GO analysis showed that the down-regulated mRNAs were involved in small molecule metabolic process, oxidation–reduction, O-glycan processing, transmembrane transport, and DNA-dependent transcription (Fig. 3a). The up-regulated mRNAs were found to be involved in the GO items “response to virus,” “immune response,” “regulation of transcription,” “DNA templated,” and “multicellular organism development” categories (Fig. 3b).

### Pathway analysis and lncRNA target pathway network

**Fig. 4:**
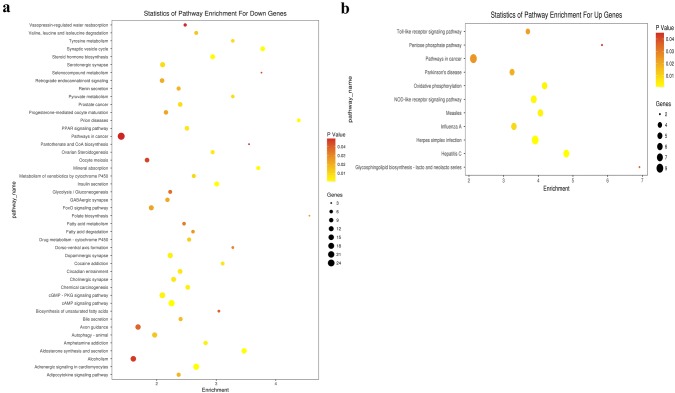
Pathway enrichment and lncRNA target pathway network. **a** Pathway enrichment according to the down-regulated mRNAs in ApoE^−^/^−^ mice. **b** Pathway enrichment according to the up-regulated mRNAs in ApoE^−^/^−^ mice

**Fig. 5:**
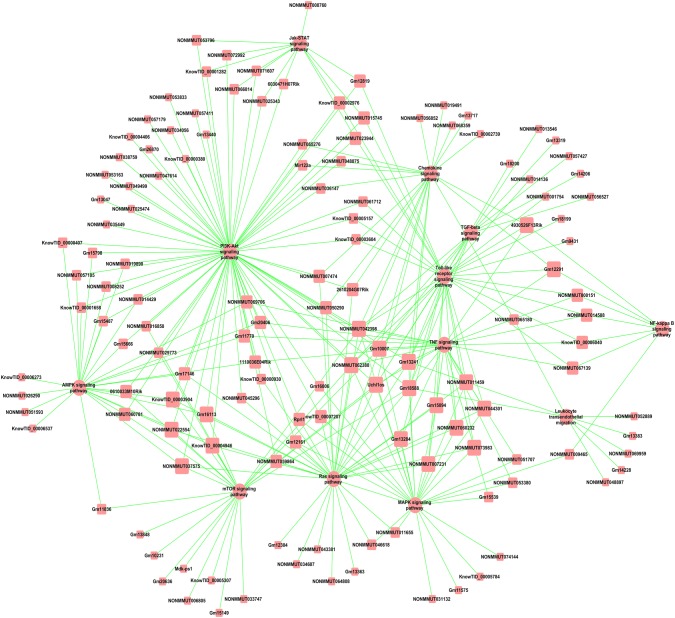
lncRNA target pathway network analysis. Square boxes represent lncRNAs, and circles represent signaling pathways. Degree means the contribution of one lncRNA to the pathway, and the main pathways are in the core of the network

Pathway analysis was performed using the KEGG (http://www.genome.jp/kegg/), and the hypergeometric distribution relationship among the differentially expressed genes was calculated to reveal which pathways might be related to the differentially expressed genes. The KEGG pathway analysis indicated down-regulation of multiple signaling pathways in ApoE^−^/^−^ mice, including olfactory transduction, PI3K-Akt signaling, neuroactive ligand–receptor interaction, endocytosis, cytokine–cytokine receptor interaction, MAPK signaling, viral carcinogenesis, Ras signaling, Epstein–Barr virus infection, and regulation of actin cytoskeleton (Fig. 4a). At the same time, several signaling pathways that play important regulatory roles in the occurrence and development of atherosclerosis were up-regulated in ApoE^−^/^−^ mice (Fig. 4b). To investigate the role of lncRNAs in regulating signaling pathways involved in atherosclerosis, we constructed a lncRNA target pathway network and found that lncRNAs play important roles in the pathogenesis of atherosclerosis via many signaling pathways including PI3K-Akt signaling, chemokine signaling, Jak-STAT signaling, leukocyte transendothelial migration, transforming growth factor (TGF)-beta signaling, Toll-like receptor signaling, MAPK signaling, mammalian target of rapamycin (mTOR) signaling, nuclear factor-kappa B (NF-κB) signaling, tumor necrosis factor (TNF) signaling, and Ras signaling (Fig. 5). Of note, the PI3K-Akt signaling pathway seemed to play a pivotal role in atherosclerosis.

### Co-expression network

**Fig. 6:**
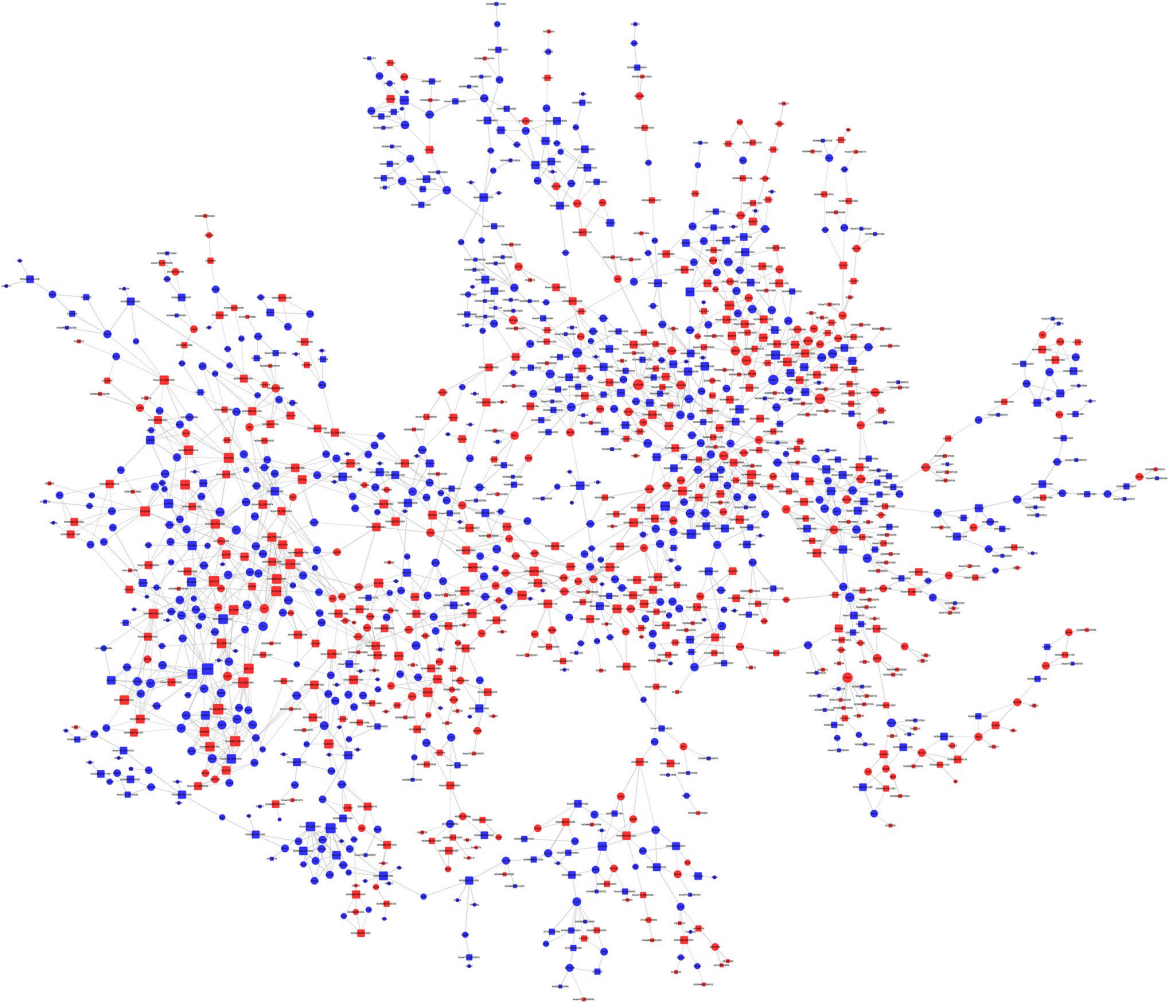
lncRNA–mRNA co-expression network analysis. Box nodes represent lncRNAs, and cycle nodes represent mRNAs. Red color indicates up-regulation, and blue color indicates down-regulation. The size of the node’s area represents the centrality value. (Color figure online)

**Fig. 7:**
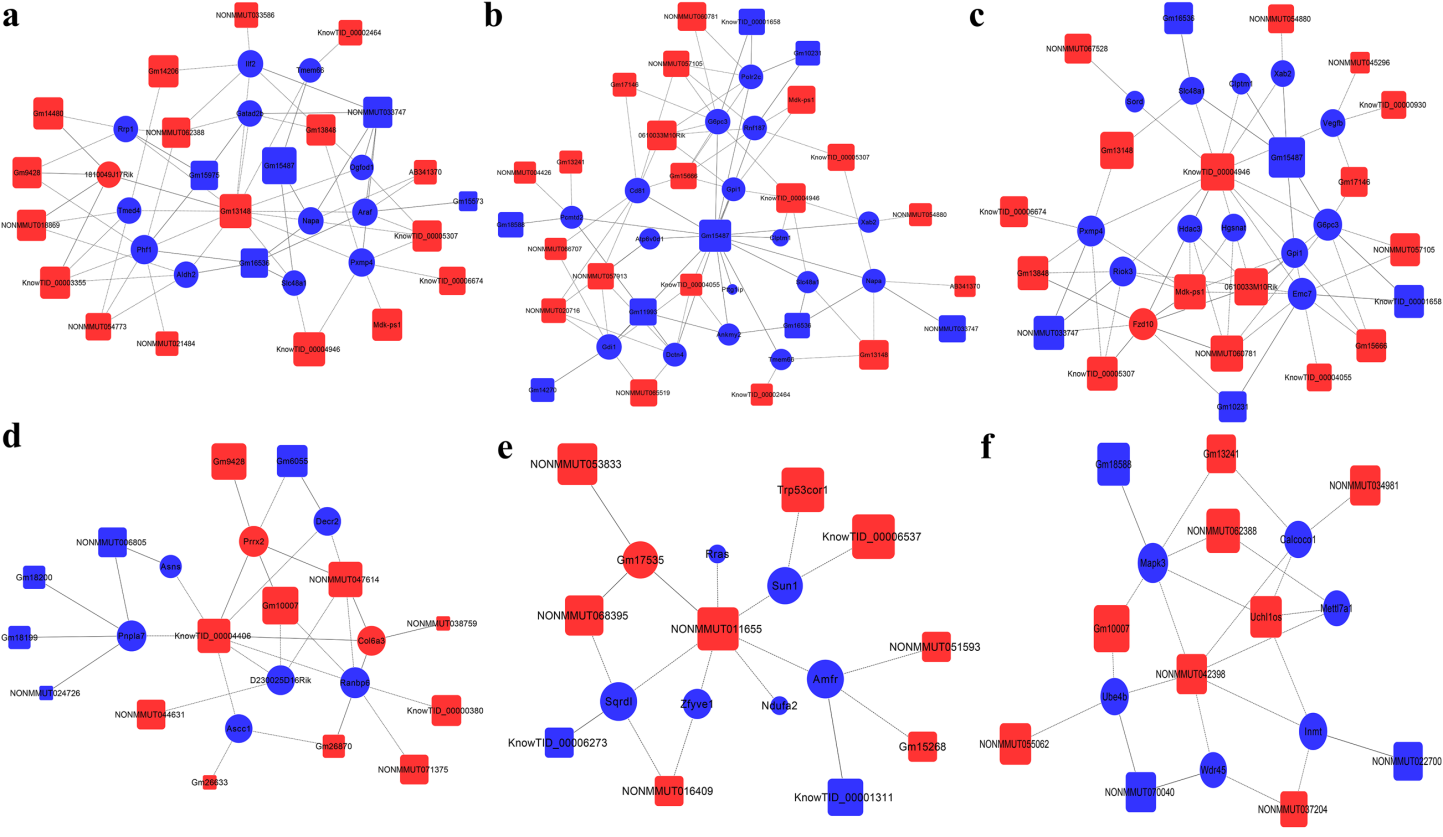
Representative co-expression networks for Gm13148 (**a**), Gm15487 (**b**), KnowTID_00004946 (**c**), KnowTID_00004406 (**d**), NONMMUT011655 (**e**), and NONMMUT 042398 (**f**) with the target genes

A lncRNA–mRNA co-expression network was constructed to identify the functions of the differentially regulated lncRNAs and mRNAs in ApoE^−^/^−^ mice compared to the control mice. For each lncRNA–mRNA pair, the co-expression degree was calculated to measure the centrality of a lncRNA in genes or networks, and the significance level was evaluated by the *p* value. Core genes were determined by the degree of difference between the two groups (Fig. 6 ). Representative co-expression networks of Gm13148, Gm15487, KnowTID_00004946, KnowTID_00004406, NONMMUT011655, and NONMMUT 042398 with the target genes are shown in Fig. 7.

### QRT-PCR validation

**Fig. 8:**
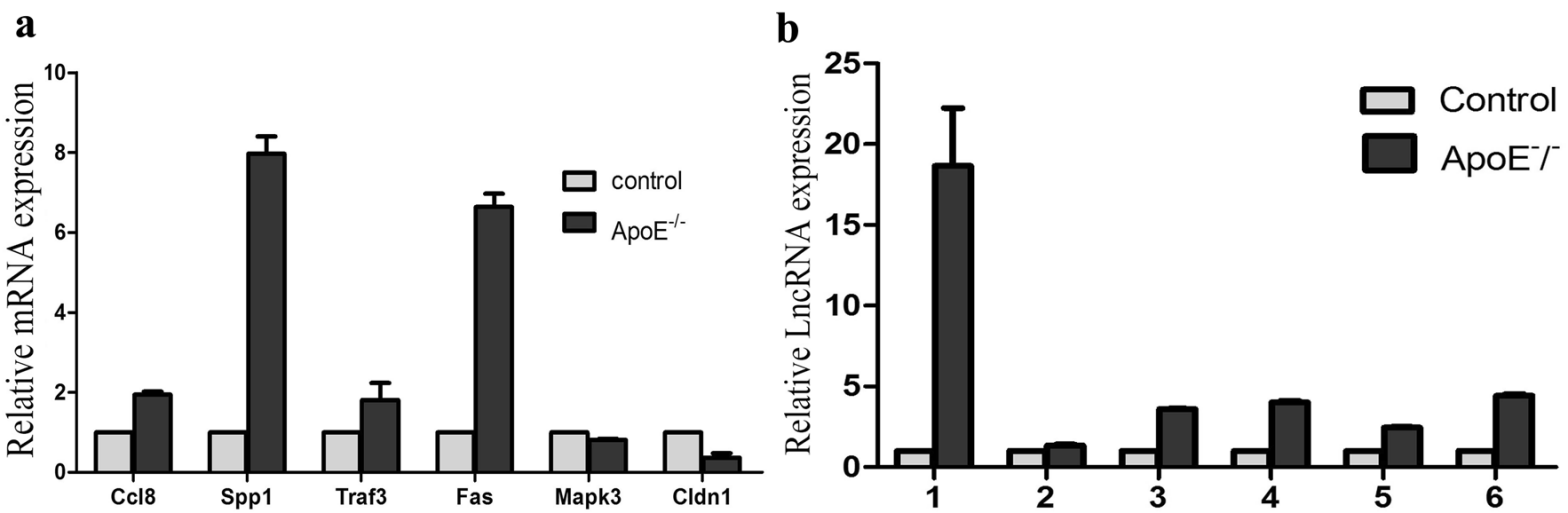
QRT-PCR validation of the altered mRNA/lncRNA expression in the aorta of ApoE^−^/^−^ mice compared to that in the control mice. **a** Six differentially expressed mRNAs identified by microarray analysis were selected for QRT-PCR validation in the aortas from ApoE^−^/^−^ and control mice. **b** Six differentially expressed lncRNAs identified by microarray analysis were selected for QRT-PCR validation in the aortas from ApoE^−^/^−^ and control mice. (1: NONMMUT000151, 2: NONMMUT042398, 3: Gm13241, 4: NONMMUT019491, 5: NONMMUT001754, 6: NONMMUT051707). (*p < 0.05)

To validate the expression patterns revealed by the microarray analyses, the expression levels of the lncRNAs and mRNAs selected above were analyzed by QRT-PCR. Consistent with the microarray results, QRT-PCR analysis showed that CC18, SPP1, TRAF3, and FAS were significantly up-regulated, while MAPK3 and CIDN1 were significantly down-regulated in the aorta of ApoE^−^/^−^ mice compared to their expression levels in the aorta of control mice (Fig. 8a); except for NONMMUT042398, all other selected lncRNAs were significantly up-regulated in the aorta of ApoE^−^/^−^ mice compared to their expression levels in the aorta of control mice (Fig. 8b).

## Discussion

In this study, we successfully established an ApoE^−^/^−^ mice atherosclerosis model fed with HFD for 12 weeks. The aorta of ApoE^−^/^−^ mice showed obvious atherosclerotic plaques compared with that of the control mice, which was confirmed by oil red O staining. The expression profiles of lncRNAs and mRNAs in aorta revealed significant differential expression of lncRNAs and mRNAs, which will provide basic research resources for a more complete understanding of ApoE^−^/^−^ mice as models of hyperlipidemia, atherosclerosis, fatty liver, diabetes, and other arterial diseases. In addition, the differentially expressed genes in ApoE^−^/^−^ mice and their functional annotation will provide an important theoretical basis for further studies of this animal model.

The reduction of oxidation–reduction, steroid metabolism and transport, and steroid biosynthesis indicates the body’s self-regulation response under high-fat stress. The role of immune mechanisms in the progression of atherosclerosis has been well established [23]. Moreover, innate immune response is regulated at different stages of atherosclerosis, while adaptive immune response is mainly regulated by the balance of regulatory T cells and effector T cells [24]. It is well accepted that there exists an inflammatory response during atherosclerosis from its inception to terminal manifestation [25]. Mononuclear phagocytes contribute to all stages of this disease, illustrating the link between inflammation and atherosclerosis [26]. Consistent with these findings and the results of Chen et al. [27], our GO enrichment analysis suggests that the differentially expressed mRNAs and lncRNAs in ApoE^−^/^−^ mice are involved in oxidation–reduction, steroid metabolism and transport, steroid biosynthesis, regulation of blood pressure, cholesterol metabolism, regulation of the force of heart contraction, virus infection response, and immune response. Our results indicate potential functions of differentially expressed mRNAs and lncRNAs in lipid metabolism and atherosclerosis development. Moreover, the up-regulation of immune response and immune globulin production by altered expression of mRNAs and lncRNAs in ApoE^−^/^−^ mice reflects the important role of the immune response in the process of atherosclerosis.

The progression of atherosclerosis is associated with dysfunction of vascular endothelial cells, immune metabolism of monocytes macrophages, and excessive proliferation of vascular smooth muscle cells [28–30]. Tang et al. showed that miR-126 mimics rescue ox-LDL-impaired autophagy flux by inhibiting PI3K/Akt/mTOR signaling [31]. Lv et al. reported that miR-155 works as a regulator of endothelial function under oxidated-low-density lipoprotein (ox-LDL) stress, which promotes autophagy in human endothelial cells by repressing Rheb/mTOR signaling [32]. Chemokines are crucial players in directing the movement and activity of leukocytes in homeostasis, immune surveillance, and inflammation. Many studies have demonstrated the important roles of chemokines and chemokine receptors in the development of atherosclerosis [33–35]. Our pathway enrichment analysis showed that these abnormally expressed mRNAs were closely related to the PI3K-Akt signaling, cytokine–cytokine receptor interaction, mTOR signaling, chemokine signaling, JAK-STAT signaling, and MAPK signaling pathways, most of which are closely related to the progression of atherosclerosis. In addition, many studies have demonstrated critical roles of specific lncRNAs in atherosclerosis [36]. Pan et al. showed that lncRNA H19 promotes atherosclerosis by increasing the activity of the MAPK and NF-κB signaling pathways and suppressing apoptosis [37]. Yao et al. reported that lncRNA ENST00113 promotes cell proliferation, survival, and migration by activating the PI3K/Akt/mTOR signaling pathway in atherosclerosis [38]. In the present study, a lncRNA target pathway network was constructed to explore the role of lncRNAs in the signaling pathways of atherosclerotic progression. These results provide insight into the pathogenesis of atherosclerosis and potential diagnostic and therapeutic lncRNA biomarkers for atherosclerosis.

Our co-expression network analysis revealed 2668 interactions containing 1144 lncRNAs and 935 mRNAs. Further QRT-PCR showed that CC18, SPP1, TRAF3, and FAS were significantly up-regulated while MAPK3 and CIDN1 were significantly down-regulated in the aorta of ApoE^−^/^−^ mice. Altered expression of lncRNAs Gm9431, NONMMUT019491, NONMMUT 000151, NONMMUT051707, Gm13241, and KnowTID_00005157 as revealed by microarray was also validated by QRT-PCR. Cldn1 is a member of the claudin family of proteins, which are integral membrane proteins and components of tight junction strands and serve as a physical barrier to prevent solutes and water from passing freely through the paracellular space between epithelial and endothelial cell sheets. The down-regulation of Cldn1 suggests endothelial dysfunction, which may affect the barrier function of the vascular endothelium. Ccl8 belongs to the chemokine family that plays an important role in the chemotaxis of monocytes, mast cells, T cells, and others [39–42]. Interestingly, Ccl8 is highly expressed and co-expressed with lncRNA NONMMUT019491, suggesting a role for Cc18 in the chemotaxis and progression of monocytes during atherosclerosis. Spp1 is a pro-inflammatory cytokine that orchestrates cell recruitment and cardiac architecture. Caballero et al. reported that spp1^−^/^−^ mice have a low heart-to-body ratio as well as reduced inflammatory pathology, CCL5 expression, myocyte size, and fibrosis in cardiac tissues, leading to the proposal that endogenous Spp1 is a key player in the pathogenesis of chronic Chagas heart disease [43]. Our observation of Spp1 overexpression in ApoE^−^/^−^ mouse model indicates that Spp1 may accelerate the process of atherosclerosis by inducing inflammation. In addition, Spp1 is co-expressed with KnowTID_00005157, NONMMUT061712, and KnowTID_00003604, revealing a relationship between lncRNAs and atherosclerosis. Khan et al. found that Mapk3 might play key roles in obesity [44]. We found that Mapk3 is involved in many important signaling transduction pathways in atherosclerosis progression such as the Toll-like receptor, TGF-β, PI3K-Akt, MAPK, and mTOR signaling pathways. Moreover, lncRNAs NONMMUT042398, Gm18588, Gm13241, NONMMUT062388, and Gm10007 were co-expressed with Mapk3. Accumulating evidence demonstrates the regulatory roles of lncRNAs in the pathogenesis and progression of atherosclerosis. Chen et al. demonstrated that knockdown of lncRNA GAS5 reduces the apoptosis of THP-1 cells treated with ox-LDL [42]. Wu et al. identified lincRNA-p21 as a therapeutic target in atherosclerosis [45]. Zhan et al. found that RP11-714G18.1 may play an atheroprotective role by inhibiting vascular cell migration via the RP11-714G18.1/LRP2BP/MMP1 signaling pathway [46]. Our findings of the extensive co-expression of lncRNAs with mRNAs provide a reference for further study of the specific molecular mechanism of atherosclerosis.

## Conclusions

In summary, we profiled the expression of lncRNAs and mRNAs in the aorta of an ApoE^−/−^ mouse atherosclerosis model. Further GO analysis and pathway enrichment analysis revealed the major changes in genes and related signaling pathways during atherosclerosis. This study may provide the basis for further investigation of the roles of lncRNAs during atherosclerosis and for identification of biomarkers for the diagnosis, treatment, and prognosis of atherosclerosis.

## Electronic supplementary material

Below is the link to the electronic supplementary material.

Supplementary material 1 (XLSX 1986 kb)
